# Levels of C-reactive protein, creatine kinase-muscle and aldolase A are suitable biomarkers to detect the risk factors for osteoarthritic disorders: A novel diagnostic protocol

**DOI:** 10.22088/cjim.10.1.25

**Published:** 2019

**Authors:** Apurba Ganguly

**Affiliations:** 1. Founder and Chief Scientific Officer, OPTM (Organic Phyto Therapeutic Method) Research Institute, Kolkata, India

**Keywords:** Osteoarthritic disorders; Diagnostic protocol; Biochemical markers; Risk factors; Degenerative changes

## Abstract

**Background:**

C-reactive protein (CRP), creatine kinase-muscle (CK-MM) and aldolase A (AldoA) levels are predicted to be realistic biomarkers of osteoarthritic disorders (OADs). The objective of the study was to evaluate the levels of CRP, CK-MM, and AldoA and determine their correlations

with risk factors such as inflammation, muscle degeneration, and skeletal muscle damage for OADs.

**Methods:**

Baseline data from 297 patients, average aged 60.17±7.19 years, suffering with OADs for 5.75±1.32 years and 315 participants, average aged 58.96±8.63 years, without OADs were collected in this cross-sectional study. Separate analyses were performed for the participants with and without OAD symptoms confirming with X-ray or MRI. Blood CRP, CK-MM, and AldoA levels were estimated. The receiver operating characteristic (ROC) curves, their respective 95% confidence intervals (CIs), and significance values were compared between participants with and without OADs to identify the risk factors for OADs in relation to biomarkers.

**Results:**

In patients with OADs, who exhibited degenerative changes on musculoskeletal joints and risk factors based on the elevated levels of CRP, CK-MM, and AldoA having their mean±SD values, 7.22±6.09 mg/L,135.2±78.56 U/L and 8.09±2.15 U/L, respectively. Their respective values of areas under the curves (AUC) of ROC curves were 0.76, 0.68 and 0.91 respectively, of which all exhibited highly significant differences (p<0.0001) compared with the control subjects.

**Conclusion:**

It is concluded from the results that the elevated levels of studied biomarkers represent the predictive above-mentioned risk factors during OADs; therefore, monitoring CRP, CK-MM and AldoA levels may be an effective diagnostic method confirming with radiological imaging.

Osteoarthritic disorder (OAD) is a chronic arthropathy characterized by the potential loss of joint cartilage as well as bone hypertrophy (osteophyte formation), connective tissue damage, pain symptoms, joint effusion, restricted movement of joints, tenderness and crepitus in the joints; OAD is also an inflammatory disease (1-5). OADs usually develop in the knee, ankle, and hip joints and in spinal vertebrae (6-7). Several diagnostic protocols based on radiology, anatomy, biochemistry, and hematologies, among others, have already been established (8-12). 

Numerous biochemical parameters are abnormally altered in various tissues, such as blood, cartilage, bones and synovial fluid, in male and female adults with osteoarthritis (12-19). Generally, serum C-reactive protein (CRP) levels are elevated during inflammation (20). Creatine kinase-muscle (CK-MM) levels are increased due to muscular dystrophy, connective tissue damage, etc. (21-22). Aldolase A (AldoA) is an enzyme expressed in a variety of tissues, with the highest levels in muscle, and serum Aldo.A levels are elevated in response to skeletal muscle disease, polymyositis, dermatomyositis, muscular dystrophy and inflammatory muscle disease (23). Previous studies have utilized an individual research approach for different diseases (20-22, 24-26) but have not studied in combined affected the serum levels of these markers in OADs.

Serum CRP levels have been documented as a potent marker for inflammation during OADs (27). Serum CK-MM levels are reported to be a biochemical marker of muscle and connective tissue damage (28), and elevated serum AldoA levels have been considered a biomarker for skeletal muscle damage (29). However, a combined approach using all of these markers to detect risk factors for OADs from control and experimental groups has not been attempted.

The liver secretes CRP into circulation during infection and/or injury, subsequently causing inflammation. CRP was first discovered by Tillett and Francis (30) and has been studied in laboratory and clinical research by other researchers (28-30). OADs may progress when levels of biochemical markers, such as CRP (< 6 mg/L), exceed standard values. 

On the other hand, there is another non-specific inflammation marker which is the erythrocyte sedimentation rate (ESR), the rate at which red blood cells fall is measured in a period of an hour (31). The common method of measuring ESR is via Westerngren method or Wintrobe method. The normal range of ESR is <22 mm/hr for men and <29 mm/hr for women. There are multiple causes of a high ESR test result, which is commonly used to identify the inflammation rate during infection, rheumatoid disease, anemia, lymphoma, multiple myeloma, pregnancy, temporal arteritis, thyroid disease, Waldenstrom macroglobulinemia etc. (32) but not in OADs. Levels of serum CK-MM can be estimated using the well-established method of measuring CK-MM isoforms (33-36); CK-MM is also expressed in several muscle damage disorders. The standard concentration of CK-MM is <168 U/L. Researchers have also documented the procedure for estimating AldoA levels (reference value is < 7.6 U/L) in patients with various diseases, such as skeletal muscle damage (28, 37-38).

The author has already established in the previous study that the degeneration in the lumber region always affects the bilateral knee joints with degenerative changes and the phenomenon is always true in reverse (9). Therefore, OADs are developed simultaneously in other multiple musculoskeletal joints such as bilateral knee joints, lumbar spine, hip joints etc. along with painful joint effusion (inflammation/swelling), tenderness, crepitus and restricted movement in the joints. All the bones and their joints are connected with muscles through tendons. Thus, the primary cause of joint pain is the damage of connective muscles along with inflammation resulting to which there is restricted movement of the joints with stiffness and decreased range of motion. Moreover, cartilage protects the joints by absorbing the pressure and shock created when we move and put stress on them. The primary diagnosis of OAD by physical examination, of joints is warmness, redness, crepitus, and limited range of motion in the joints. After physical examination to confirm the OADs on multiple joints, the imaging scans such as CT-scan or x-ray or MRI are done to produce an image of bones in case of x-rays for multiple joints which has limitations of too much radiation in the body. Also, restricted to pregnant patients but not identifying the quantum of inflammation, muscle degeneration and skeletal muscle damage and in case MRI produces images of bones, ligaments, cartilage and synovits with some limitations such as certain metal objects implanted in the body viz namely: pacemakers, prosthetic joints, rods and certain tattoos and also restricted to overweight, very tall and claustrophobic patients. Moreover, MRI is very costly, and it is to be used for multiple joints to identify the damage made during OADs. 

There are other common blood tests such as anti-cyclic citrullinated peptide (anti-CCP), rheumatoid factors and antinuclear antibody (ANA) to diagnose osteoarthritis (OA) but these are very costly and time dependent tests, as well as do not firmly quantify the damages made in the muscle and tissue levels during OADs due to inflammation, muscle degeneration, bone erosion, inflammatory muscle disease, and skeletal muscle damage causing to develop muscular dystrophy. Moreover, the author has already developed a unique deranged anatomical measuring protocol to identify quantum of damage of muscles and tissues and also established that with the help of another biochemical test methods to identify the quantum of damages in bone level such as levels of calcium, phosphorus, ratio of calcium-phosphorus, 25-OH vitamin D, and intact parathyroid hormone (iPTH) during OADs (39-41). Again, the author has previously established that the elevated levels of CRP, CK-MM and Aldolase A can be normalized during OADs with the help of certain specific phytotherapeutic treatment protocol (42). 

The present study attempted for the first time to identify that biochemical markers namely; CRP, CK-MM and Aldo A are the risk factor for OADs. The combination of the above-mentioned blood markers for detecting OADs may be an exclusive approach because researchers identified the disease with single marker only and no one attempted earlier with these combinations. Radiological images such as x-ray or CT-scan or MRI as the only diagnostic tools for detecting OADs in advance stages and not in early progressive stages have many limitations and very expensive as well. Moreover, for the detection of OADs, many other methods may be alternatively adopted such as physical examination, serum anti-CCP and ANA, rheumatoid factors, ratio of serum calcium to phosphorus, and iPTH for specific area of study. 

But through the investigation of the above-mentioned biochemical parameters such as CRP, CK-MM and Aldo A from the patient’s serum can be identified accurately, the quantum of inflammation, muscle degeneration, skeletal muscle damage, which are the key predictive factors of OADs, quickly and in affordable low cost in the early stage of OAD where there is no pain syndrome or discomfort or deformities observed in the joints much before focusing in the radiological images. Evaluating the data using specific statistical tests, including ROC curves analysis. Therefore, the present study has portent the novelty concepts for diagnosis of OADs into the relevant biomedical categories. 

## Methods

**Recruitment of participants: **A total of 1017 participants, aged 40 to 80 years old who were treated at OPTM Health Care (P) Ltd. Centers in Kolkata, Delhi and Mumbai, India from January 2016 to March 2017 were evaluated in this study. The study protocol was evaluated and approved by the organic phyto therapeutic method (OPTM) Research Institute Ethics Committee. The institute is registered with the government. An Institutional Review Board-approved consent form for the physical examinations, blood sample collections and cervical spine, lumbar spine, hip and knee joint images (x-rays, CT scan or MRI) required for the study was signed by all patients in the first phase of the screening procedure.

**Exclusion criteria: **Four hundred and five of 1017 participants were excluded for having other pathological conditions that could explain the existing symptoms, such as rheumatic diseases, osteochondritis diseases, inter-articular fractures, congenital dysplasia, radicular syndrome, joint symptoms caused by malignant tumors, Baker’s cyst, Perthes disease, Plica syndrome, dermatomyositis and polymyositis diseases, iliopectineal or trochanteric bursitis bone and joint infectious diseases and ischemic bone necrosis. The following additional exclusion criteria were considered: patients with multiple drug dependence; a history of cancer, including carcinomatosis and granulocytic leukemia; patients with cuts, wounds, or any type of chronic skin disease; a history of severe neurological diseases; a history of chronic liver, kidney and heart diseases; and patients who did not agree to a physical evaluation and/or attend weekly follow-up visits.

**Study design: **After evaluating the exclusion criteria, 315 of the remaining 612 subjects with no complaints of pain or visual inflammation and no signs of OADs in the cervical region, lumber region, hip joints, knee joints or any parts of the body, as evidenced by x-ray, CT scan or MRI, were considered the control subjects. The remaining 297 subjects who had OADs in any region, jointly or singly, according to the radiological analysis and complained of pain and visual inflammation were considered the experimental group. The baseline demographic characteristics of all patients are presented in table 1. Co-morbidities were also assessed using the Charlson co-morbidity index and methods described by Katz et al. (43) and Singh et al. (44).

**Statistical analysis: **Continuous variables, such as serum CRP, CK-MM, and AldoA levels, in the OAD cohort are expressed as the mean, standard deviation (SD), and 95% CIs of differences and were compared between the experimental and control groups using the Mann-Whitney U test, as the data do not follow a normal distribution. Non-parametric receiver operating characteristic (ROC) curve analysis was performed to evaluate the accuracy of various measurements in predicting the experimental group, as indicated by the area under the curve (AUC). The AUCs for CRP, CK-MM, and AldoA were calculated separately. ROC curves indicate the relationship between the true positive (sensitivity) and false positive (1- specificity) cases and were constructed for CRP, CK-MM and AldoA. An AUC of 1 indicates a unique test with 100% sensitivity and specificity, whereas an AUC of less than 50% indicates that the diagnostic test is less useful. The statistical software IBM SPSS (Version 20) was used for all statistical analysis. An alpha level of 5% was established i.e., a p-value less than 0.05 was considered statistically significant.

**Evaluation of specific biochemical parameters in blood: ** A 5-ml blood sample was collected in a plain vial from each subject with or without OADs. Blood samples were then centrifuged at 1000×g for 10 min at 4^o ^C to obtain serum. Finally, the serum was used to analyze CRP, CK-MM, and AldoA levels in both experimental and control subjects. The biomarkers were rigorously analyzed. Each test for each experimental and control subject has been rechecked by the BS-240 Mindray fully automated biochemistry analyzer before reporting the final test result. 

CRP levels (mg/L) were quantitatively assessed using a CRP LEIT kit and the latex-enhanced turbidimetric immune assay (Agappe Diagnostics Limited, India) at a wavelength of 578 nm. The kit was developed based on the methods reported by Tillett and Francis (27), Spector and Hart (45) and Rifai et al. (46). CK-MM (U/L) levels were quantitatively assessed using a Creatine Kinase- MM kit (CK-MM/CPK-MM/CK-3) and an immunoassay (Aalto Scientific Limited, USA). The kit was developed based on the methods reported by Walker (47). AldoA levels (U/L) were quantified using an ALDOLASE (ALS) RX MONZA AD 189 kit (Randox Laboratories Ltd, Antrim, UK) based on a photometric assay at a wavelength of 340 nm. The kit was developed according to the method reported by Thompson (48). The subjects suffering from OADs with inflammation, muscle degeneration and skeletal muscle damage were studied to identify a specific biochemical parameter, such as CRP, CK-MM, and AldoA levels, in the affected population. All blood tests were conducted under the supervision of the Chief Biochemist and the Chief Pathologist in the Galaxy Medical Centre, an ISO 9001: 2015 certified lab with Registration No. L/004-(05)-15/0129 under the W.B Clinical Establishment Act, 1950.

**Evaluations of the mean, standard deviation and significance value and the ROC curves analysis: **The mean, standard deviation, mean difference (MD), 95% confidence intervals (CI) and their significance values (p-values) were evaluated and ROC curves analyses were performed for the three biomarkers among 297 subjects with OADs and 315 subjects without OADs. While analyzing the ROC curves, the sensitivity and specificity for each biochemical parameter were calculated and thereafter, the accuracy, positive predictive value (PPV) and negative predictive value (NPV) were evaluated with the help of following formulae:

Prevalence = (true positive + false negative)/ (true positive + false positive +false negative +true negative);

Accuracy= sensitivity x prevalence + (specificity) x (1-prevalence); positive predictive value (PPV) = (sensitivity x prevalence)/[sensitivity x prevalence + (1-specificity) x(1-prevalence)];

Negative predictive value (NPV) = specificity x (1-prevalence)/ [(1-sensitivity) x prevalence + specificity x (1-prevalence)]. 

**Evaluation of correlation coefficients between two biomarkers: **To determine the predictive values of the three biomarkers in patients with OADs, the correlation coefficients between two biomarkers were separately evaluated for OAD and non-OAD participants.

## Results

A total of 297 patients aged 60.17±7.19 years those who were suffering with OAD for 5.75±1.32 years and 315 patients aged 58.96±8.63 years without OAD symptom confirmed by x-ray or MRI. 

Baseline demographic characteristics of all patients for both experimental and control groups who were not being treated by oral medications; injections; massage with any type of herbal gels; and any type of alternative interventions or treatments for diminishing pain or inflammation, for muscle relaxation, or to improve the skeletal muscles during the last four weeks and did not undergo arthrocentesis within three months prior to the blood tests are shown in table 1. The risk factors and values of the biochemical markers, such as CRP, CK-MM, and AldoA were evaluated using the serum samples of experimental subjects suffering from OADs and control subjects without OADs.

Table 2 A shows that the mean± SDs of CRP, CK-MM and AldoA levels for 297 subjects with OADs and that these differences were highly significant (P<0.001). Compared to the 315 subjects without OADs. 

**Table 1:** Demographic data and baseline characteristics of subjects

**Characteristics**	**Experimental group**	**Control group**
No of subjects	297	315
Age (yrs), (mean±SD)	60.17 (7.19)	58.96 (8.63)
BMI (kg/m²) (mean±SD)	29.94 (3.31)	28.42 (3.38)
Females	193 (65%)	231 (73%)
Period of suffering (yrs), (mean±SD)	5.75 (1.32)	**-**
**Indian ethnic group (%)**		
Bengali	65 (22)	71 (22)
Gujrati	34 (11)	35 (11)
Marwaree	41(14)	43 (14)
Marathi	29(10)	33 (10)
Tamil	37 (12)	37 (12)
Punjabi	38 (13)	41 (13)
Shindhi	29 (10)	31(10)
North East India	24 (8)	24 (8)
**Food habit (%)**		
Vegetarian	181 (61)	205 (64)
Non - vegetarian	116 (39)	115 (36)
**Analysis of radiological reports (%)**		
Unilateral knee OAD* with osteophytes	63 (21)	**-**
Bilateral knee OAD with osteophytes	55 (19)	**-**
OAD changes in cervical vertebrae	39 (13)	**-**
OAD changes in lumbar vertebrae	51 (17)	**-**
Unilateral OAD in hip joint with osteophytes	27 (9)	**-**
Bilateral OAD in hip joints with osteophytes	28 (10)	**-**
Unilateral OAD in shoulder joint	23 (8)	**-**
Bilateral OAD in shoulder joints	11 (3)	**-**
Unilateral OAD in ankle joint	8(2)	**-**
Bilateral OAD in ankle joints	7(2)	**-**
**Work status (%)**		
Employed fulltime	78 (26)	93 (30)
Employed part time	21 (7)	25 (8)
Housewife / Homemaker	72 (24)	83 (26)
Retired	45 (16)	42 (13)
Self employed	81 (27)	72 (23)
**Multiple complaints (%)**		
Constipation	68 (23)	80 (25)
Acidity & reflux	72 (24)	45 (14)
Insomnia	66 (22)	62 (20)
Varicose vein	34 (11)	32 (10)
Urinary incontinence	43 (14)	17 (5)
Crepitus during knee flexion	32 (11)	-
Morning stiffness (<30 minute)	37 (12)	-
**Measures taken to diminish pain (%)**		
Using knee caps	69 (23)	**-**
Using a lumbar belt	49 (16)	**-**
Using a collar belt	35 (12)	**-**
Using a stick/walker	34 (12)	**-**

* OAD: Osteoarthritic disorder

**Table 2A:** Statistical analysis of the mean, standard division (SD) and 95% confidence interval (CI) for 297 OAD subjects and 315 subjects without OAD

**Biochemical parameters**	**Control group**	**Experimental group**	**P-value**	a **MD**	**95% CI of difference**	
	**Mean (SD)**	**Mean (SD)**			**Lower**	**Upper**
CRP (mg/L)	3.54 (1.45)	7.22 (6.09)	<0.001	-3.68	-4.37	-2.99
CK-MM (U/L)	91.44 (38.71)	135.22 (78.56)	<0.001	-43.78	-53.53	-34.03
AldoA (U/L)	5.26 (1.21)	8.09 (2.15)	<0.001	-2.81	-3.09	-2.53

a : Mean difference

**Table 2B:** Analysis of receiving operating characteristic (ROC) curves for 297 OAD subjects and 315 subjects without OAD

**Biochemical parameters**	**Receiving operating curve analysis**			
	**Area under the curve (AUC)**	**p-value**	**95% CI of AUC**	
			**Lower**	**Upper**
CRP (mg/L)	0.76	<0.001	0.72	0.80
CK-MM (U/L)	0.68	<0.001	0.64	0.72
AldoA (U/L)	0.91	<0.001	0.88	0.93

When analyzing the ROC curves, the optional cutoff points were considered as <6, <168 and <7.6 for CRP, CK-MM and Aldo A respectively. The analyses of ROC curves in respect of CRP, CK-MM and Aldo A for 297 OAD subjects and 315 non-OAD subjects with all significant values (p<0.001) are shown in table 2B. The analyses of sensitivity, specificity, positive predictive value (PPV), negative predictive value (NPV) and diagnostic accuracy are shown in the table 3. 

**Table 3:** Analyses of Sensitivity, Specificity, PPV, NPV and diagnostic accuracy

**Parameter**	**Sensitivity (%)**	**Specificity (%)**	**PPV** * **(%)**	**NPV** * **(%)**	**Diagnostic accuracy ** **(%)**
CRP	50.51	98.41	96.77	67.83	75.16
CK- MM	48.82	78.73	68.40	62.00	64.22
ALDO A	81.82	89.21	87.73	83.88	85.62

* PPV: Positive predictive value, NPV: Negative predictive value

**Figure 1:**
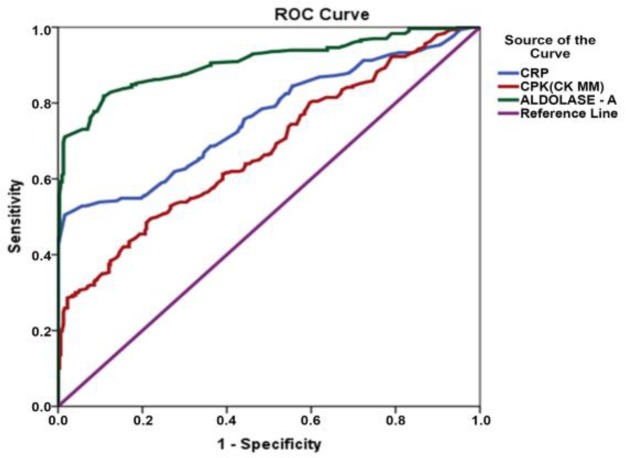
Receiver operating characteristic curves for C-reactive protein (CRP), creatine kinase-muscle (CK-MM) and aldolase A (AldoA). The area under the curve was 0.761 (95% CI = 0.723 – 0.800) for CRP, 0.677 (95% CI = 0.635 – 0.719) for CK-MM, and 0.908 (95% CI = 0.883 – 0.932) for AldoA

The ROC curves for CRP, CK-MM, and AldoA levels are depicted in Fig 1. 

The area under the curve (AUC) and 95% CIs of the AUC for the CRP, CK-MM and AldoA levels were highly significant (P<0.001) in all cases. 

Table 4 shows that the correlation coefficients between CRP and CK-MM as well as CRP and AldoA were highly significant (P=0.032 and P=0.001), whereas the R-value between CK-MM and AldoA was not statistically significant (P=0.092) for OAD patients. 

The R-values between CRP and CK-MM as well as CRP and AldoA were not significant (P=0.167 and P=0.850), but the R-value between CK-MM and AldoA was highly significant (P=0.032) for non-OAD subjects.

**Table 4:** Correlation coefficient between two biomarkers

**Biomarkers**	**Experimental group**		**Control group**	
	**R-value**	**P-value**	**R-value**	**P-value**
CRP and CK-MM	-0.124	0.032	-0.080	0.167
CRP and AldoA	0.179	0.001	-0.011	0.850
CK-MM and AldoA	0.097	0.092	0.123	0.032

## Discussion

The reference value for each biochemical parameter in adults has been established as <6 mg/L CRP, <168 U/L CK-MM and <7.6 U/L AldoA. CRP levels increase due to inflammation (20, 43-45), CK-MM levels are elevated in response to muscular dystrophy, connective tissue damage, etc. (21-22; 25), and AldoA levels increase due to skeletal muscle damage and bone erosion (23), suggesting that these markers may be risk factors for OADs. It is further indicated from the results that a significantly increasing trend was observed (p<0.001) for each parameter when compared to the subjects having OADs with the control subjects However, previous studies were conducted in separate subjects by several researchers, and no study has attempted to correlate the risk factors, such as muscular and skeletal muscle damage and inflammation, with OADs.

Researchers have investigated the relationship between AldoA levels in the circulation and levels of the anti-aldolase A autoantibody in serum, but no correlation was observed. The reactivity of anti-aldolase A autoantibody in the serum of patients with rheumatoid arthritis was evaluated using denatured and native AldoA, and this autoantibody predominantly bound to denature enzyme (23).

The ROC curve has many advantages in medical and/or biological sciences, as it has been reported by many researchers (55-60). The analysis of ROC curve is used to determine the risk factors based on biomarker data from biochemical analysis in the clinic (61). 

In statistical analysis, the probability of a positive test result for subjects with OADs is derived from the variation of test results among subjects with and without OADs; this probability is basically a conditional probability of correctly identifying subjects with OADs and is based on the sensitivity of the test in detecting the true positive fraction. Meanwhile, the probability of negative test results for control subjects without disease is the conditional probability of correctly identifying subjects without OADs, which is based on the specificity of the test for the true negative fraction. These indices are very useful for the evaluation of biochemical markers (CRP, CK-MM, and AldoA) when data are obtained from the laboratory. Positive test results identify the risks of developing inflammation, muscular degeneration and skeletal muscle damage among patients with OADs, whereas negative test results identify the probability of being healthy without any risk factors. However, from the estimates of these biochemical markers, risk factors of OADs and correlations among variables can be identified. Nonetheless, these factors are influenced by the prevalence of OADs and the risk of other diseases in subjects. The statistical analysis of ROC curve is used to detect risk factors for OADs in the present study are commonly used by other researchers investigating the prevalence of other diseases, such as cancer and infectious diseases (61, 65-67). Finally, this prediction tool is helpful in determining diagnostic accuracy (65, 68).

 An important finding from the results showed that the elevated AldoA levels is a greater risk factor for skeletal muscle damage among patients with OADs than the other two markers, CRP and CK-MM and the values were highly significant (P<0.001) for all markers. The present results clearly indicated the presence of inflammation, which was confirmed by the CRP test, and confirmed skeletal muscle damage, as indicated by the elevated AldoA levels. CK-MM levels were also elevated along with the other two biomarkers, indicating that muscular degeneration occurred in patients with OADs (20, 22-23). 

In general, OADs can be identified only in advance stage for assessing the condition of bones at musculoskeletal joints such as joint space narrowing, osteophyte formation, sclerosis and deformity of bony ends using anterior-posterior (AP) views of x-ray images on various musculoskeletal joints by four-graded scale developed by Kellgren-Lawrance (69) but definite quantum of inflammation, muscle degeneration and skeletal muscle damage affecting bone and cartilage damage of joints cannot be identified from x-ray images whereas, MRI of various musculoskeletal joints can also be assessed at advance stage by four graded scale known as Fredericton MRI classification scale which includes periosteal edema, bone marrow edema visible only on T2-weighted image or T1- and T2-weighted images and multiple focal /linear areas of intracortical signal abnormalities (70) which are very costly affairs and not affordable by common people for various individual joints to identify OADs but not at early progressive stage. Therefore, the elevated levels of CRP, CK-MM, and AldoA observed in patients with OADs have confirmed that these biomarkers are predictive risk factors that may be monitored and serve as one of the best diagnostic protocols quickly and in affordable low cost even in the early progressive stage of OAD where there is no pain syndrome or discomfort or deformities observed in the joints confirmed with x-rays or MRI images on various musculoskeletal joints only at advance stage but not in early progressive stage. Moreover, there are certain limitations for MRI imaging such as metal objects implanted in the body such as pacemakers, prosthetic joints, rods and certain tattoos and also restricted to overweight, very tall and claustrophobic patients. 

Furthermore, the author has already developed a unique deranged anatomical measuring protocol to identify quantum of damage of muscles and tissues plus other biomarkers such as calcium, phosphorus, and ratio of calcium to phosphorus and level of parathyroid hormone to identify the condition of damaged bone during OADs (39-42). Again, the author had shown earlier that the elevated levels of CRP, CK-MM and Aldolase A can be normalized, even at early progressive stage of OADs with the help of certain specific phytotherapeutic treatment protocol when significant improvements are observed in the imaging scans such as x-ray or MRI or CT-scan (41). Additionally, the result shows the high predictive values of the three biomarkers in patients with OADs when the correlation coefficients (R-values) are separately calculated between two biomarkers. 

In conclusions based on the present study, elevated levels of the biochemical markers such as CRP, CK-MM, and AldoA represent the key predictive risks factors such as inflammation, muscle degeneration, muscular dystrophy, inflammatory muscle disease and skeletal muscle damage affecting muscle bone and cartilage damage of various musculoskeletal joints; therefore, monitoring serum CRP, CK-MM and Aldolase A levels may be a novel approach for the quick detection of OADs and in an affordable low cost even at early progressive stage when there is no significant pain syndrome or discomfort or deformities observed in the x-ray or MRI images (20-23, 43-45) 

Further research has been undertaken to identify cartilage damage, to establish diagnostic tools for other OADs, specially rheumatoid arthritis, to measure pro- and anti-inflammatory cytokines such as IL-10, TNF-α, collagen 4-hydroxyproline (O-Hyp) and hyaluronic acid (synovits) both in volume and chemical compositions which are not at all possible with the help of either x-ray or MRI imaging.

**Limitation of the study**

The patients suffering from the following disorders are restricted to participate in the present study:

1. Rheumatic diseases;
2. Osteochondritis diseases;
3. Congenital dysplasia;
4. Radicular syndrome;
5. Joint symptoms caused by malignant tumors;
6. Dermatomyositis and polymyositis diseases;
7. Iliopectineal or trochanteric bursitis;
8. Ischemic bone necrosis.
9. Bone and joint infectious diseases

10. Cuts, wounds or any type of chronic skin and infectious diseases;

11. Parallel multiple drug dependence for concomitant diseases or risk conditions requiring drug treatment including psychiatric diseases etc.

12. A history of cancer, including carcinomatosis and granulocytic leukemia.

13. A history of severe neurological diseases.

14. A history of chronic liver, kidney and heart diseases.

15. Patients who did not agree to give blood sample may be due to drugs/alcohol addiction, pregnancy and such other reasons.
